# Transcriptomic profiles of *Mannheimia haemolytica* planktonic and biofilm associated cells

**DOI:** 10.1371/journal.pone.0297692

**Published:** 2024-02-08

**Authors:** Hao Ma, David P. Alt, Shollie M. Falkenberg, Robert E. Briggs, Fred M. Tatum, Michael L. Clawson, Eduardo Casas, Rohana P. Dassanayake

**Affiliations:** 1 Ruminant Diseases and Immunology Research Unit, United States Department of Agriculture, Agricultural Research Service, National Animal Disease Center, Ames, Iowa, United States of America; 2 Infectious Bacterial Diseases of Livestock Research Unit, United States Department of Agriculture, Agricultural Research Service, National Animal Disease Center, Ames, Iowa, United States of America; 3 Department of Pathobiology, College of Veterinary Medicine, Auburn University, Auburn, Alabama, United States of America; 4 United States Department of Agriculture, Agricultural Research Service, U.S. Meat Animal Research Center, Animal Health Genomic Research Unit, Clay Center, Nebraska, United States of America; Cornell University, UNITED STATES

## Abstract

*Mannheimia haemolytica* is the principal agent contributing to bovine respiratory disease and can form biofilms with increased resistance to antibiotic treatment and host immune defenses. To investigate the molecular mechanisms underlying *M*. *haemolytica* biofilm formation, transcriptomic analyses were performed with mRNAs sequenced from planktonic and biofilm cultures of pathogenic serotypes 1 (St 1; strain D153) and St 6 (strain D174), and St 2 (strain D35). The three *M*. *haemolytica* serotypes were cultured in two different media, Roswell Park Memorial Institute (RPMI) 1640 and brain heart infusion (BHI) to form the biofilms. Transcriptomic analyses revealed that the functions of the differentially expressed genes (DEGs) in biofilm associated cells were not significantly affected by the two media. A total of 476 to 662 DEGs were identified between biofilm associated cells and planktonic cells cultured under BHI medium. Functional analysis of the DEGs indicated that those genes were significantly enriched in translation and many biosynthetic processes. There were 234 DEGs identified in St 1 and 6, but not in St 2. The functions of the DEGs included structural constituents of ribosomes, transmembrane proton transportation, proton channels, and proton-transporting ATP synthase. Potentially, some of the DEGs identified in this study provide insight into the design of new *M*. *haemolytica* vaccine candidates.

## Introduction

Bovine respiratory disease (BRD) imparts significant economic costs to both beef and dairy industries [1, 2]. It has been estimated that in North America BRD complex accounts for approximately 90% of all morbidity and mortality in stocker calves, and 75% of all morbidity and 50% of all mortality in feedlots [3, 4]. Both environmental and host factors as well as interactions with other pathogens are known to increase the severity of disease caused by *Mannheimia haemolytica* [5, 6]. A mechanism for *M*. *haemolytica* to increase its resistance to antibiotic treatments and host immune defenses is to form biofilms [7, 8].

Biofilm has been described as “city of microbes” or “superbugs” and defined as assemblages of microorganisms and their associated extracellular products at an abiotic or biotic surface [8–11]. It is formed by bacterial aggregates which tightly adhere to an interface and are enclosed in an abundant self-produced matrix of extracellular polymeric substance [7, 12]. After over 50-years of biofilm study, the life cycle of biofilm development has been depicted as planktonic cells that reversibly and irreversibly aggregate and attach to biotic or abiotic materials, and accumulate through production of biofilm matrix components, then the bacterial colonies expand by growth and recruitment of surrounding cells, and finally degrade matric components and disperse cells or colonies by disaggregation and detachment [13, 14]. This model, however, does not capture the dynamic genetic variations and activities during biofilm development.

The properties of biofilm-associated cells are distinct from planktonic cells not only for the surrounding microenvironment formed by exopolysaccharide structure and heterogeneity of oxygen and nutrients, but also by slower growth and possible bacterial mutants [15–17]. Biofilm formation increases resistance to both conventional antibiotic treatment and to the hosts’ intrinsic and acquired immune system defenses [9, 18, 19]. Bacteria in biofilms undergo a higher rate of mutation than planktonic cells leading to ten-fold increase in the efficiency of transfer of plasmids containing antibiotic resistance genes when biofilms are exposed to sub-inhibitory concentration of antibiotics [20]. Using *Escherichia coli* as a model microbe, Bernier et al., 2013 showed that an auxotroph with mutations in amino acid biosynthetic genes also showed increased resistance to certain antibiotics [21].

*M*. *haemolytica* is an opportunistic pathogenic bacterium, which usually colonizes in the upper respiratory tract of normal cattle. However, *M*. *haemolytica* descends to the lower respiratory tract and contributes to BRD when the animals’ immune systems are compromised due to exposure to a variety of predisposing factors. There are 12 capsular serotypes (St) classified for *M*. *haemolytica*. Serotype 2 is commonly isolated from the upper respiratory tract in healthy cattle [22], while St 1 and 6 are frequently isolated from nasopharynx and lungs of animals affected with BRD [23–29]. However, these all three of these serotypes are known to cause pneumonia in cattle [24].

We have previously reported the multilayer three-dimensional structure of biofilm of *M*. *haemolytica* St 1 (D153 strain) cultured in Roswell Park Memorial Institute (RPMI) 1640 medium and brain heart infusion broth (BHI) [30]. Although higher amounts of biofilm biomasses were detected for St 1 in BHI as compared to RPMI, similar percentages of live bacteria were found in both media as assessed by viability stains [30]. The objective of this study was to compare biofilm and planktonic cell transcriptomic profiles of *M*. *haemolytica* serotypes (St 1, 2 and 6) grown in different culture media (RPMI vs BHI). The results revealed molecular differences of *M*. *haemolytica* biofilm and planktonic cells, and effects of culture medium on biofilm formation.

## Materials and methods

### Bacterial strains and planktonic cell growth

*Mannheimia haemolytica* St 1 (D153) [31], St 2 (D35) [32], and St 6 (D174) [33] strains isolated from pneumonic bovine lung tissue samples were maintained as frozen stocks (-80 °C) in BHI (Becton, Dickinson Co., Sparks, MD) supplemented with 10% glycerol. A loopful of frozen bacteria were spread onto trypticase soy agar supplemented with 5% defibrinated sheep blood plates (TSA II^™^, Becton, Dickinson Co.) and incubated at 37 °C in a humidified atmosphere of 7.5% CO_2_ overnight.

A single colony from each of the serotypes was transferred into individual 50 mL conical tubes containing 10 mL BHI broth, optical density was adjusted (OD_600nm_ = ~0.150) and incubated at 37 °C with constant shaking (190 rpm) for about 2 hours (mid-log phase, OD_600nm_ = 0.6; ~1 × 10^9^ colony forming units/ml [CFU/mL]).

### Biofilm formation

*M*. *haemolytica* biofilms were allowed to form in the bottom of 24-well plates as described previously [30]. Briefly, planktonic (PL) *M*. *haemolytica* serotypes grown in BHI broth for 2 hours to mid-log phase were separately diluted in BHI broth and RPMI 1640 medium (ThermoFisher Scientific, Grand Island, NY, 1:1000, ~1 × 10^6^ CFU/mL). Eight hundred μL of diluted *M*. *haemolytica* serotypes were transferred into 24-well plates (9–12 wells each serotype) and covered with lids. Plates were incubated at 37 °C in a humidified atmosphere of 7.5% CO_2_ without shaking for 24 hours for biofilms to develop.

### RNA purification

For PL bacteria, 6 mL of RNAprotect bacterial reagent (Qiagen, Valencia, CA) was directly added to 3 mL of bacterial cultures to immediately stabilize RNA, incubated at room temperature for 5 min and centrifuged for 10 min at 10,000×*g* to pellet the bacteria. The bacterial pellets were stored at -80 °C. For biofilm (BF) bacteria, after supernatants were removed, then RNAprotect diluted in phosphate-buffered saline (2:1, 0.5 mL/well) was added to each well. To maximize for RNA yield, bacteria in three wells were combined and transferred to a single microfuge tube, incubated, centrifuged, and the pellets were frozen as described for PL bacteria. Total bacterial RNA from both PL and BF were purified using a combination of enzymatic lysis (lysozyme and proteinase K) and the columns of a RNeasy Mini Kit (Qiagen). To prevent any cross-contamination, bacterial pellets from one serotype were processed at a time. Residual amounts of genomic DNA contamination in the samples were removed by on-column DNase I digestion and total RNA was eluted using RNase-free water. RNA concentration of each sample was determined by Qubit^™^ RNA broad range assay kit using a Qubit 4 fluorometer (ThermoFisher Scientific). RNA quality of each sample was determined by RNA 6000 nano chips using an Agilent 2100 bioanalyzer system (Agilent Technologies, Santa Clara, CA) in accordance with the manufacturer’s instructions. The samples with RNA integrity number (RIN) of at least 8 were used for cDNA library preparation.

### cDNA library preparation and sequencing

A total of nine RNA samples, each with five replicates from both PL and BF of the three serotypes were used for subsequent cDNA library preparation. In order to enrich bacterial mRNA, ribosomal RNA in the samples was removed by using the NEBNext^®^ rRNA Depletion Kit (Bacteria) as per manufacturer’s instructions (NEB Inc., Ipswich, MA). Purified mRNA was enzymatically fragmented, and cDNA were generated with random hexamer primers. Specific adapters were ligated to the cDNA fragments and libraries were enriched by PCR using the NEBNext^®^ Ultra^™^ II Directional RNA Library Prep Kit for Illumia^®^ in accordance with the manufacturer’s protocol (NEB inc.). cDNA libraries were sequenced using the Illumina NovaSeq 6000 Sequencing System (Illumina, San Diego, CA), and 150bp paired-end reads were generated.

### Serotype genome alignment and RNA-sequencing data analyses

**Fig 1:**
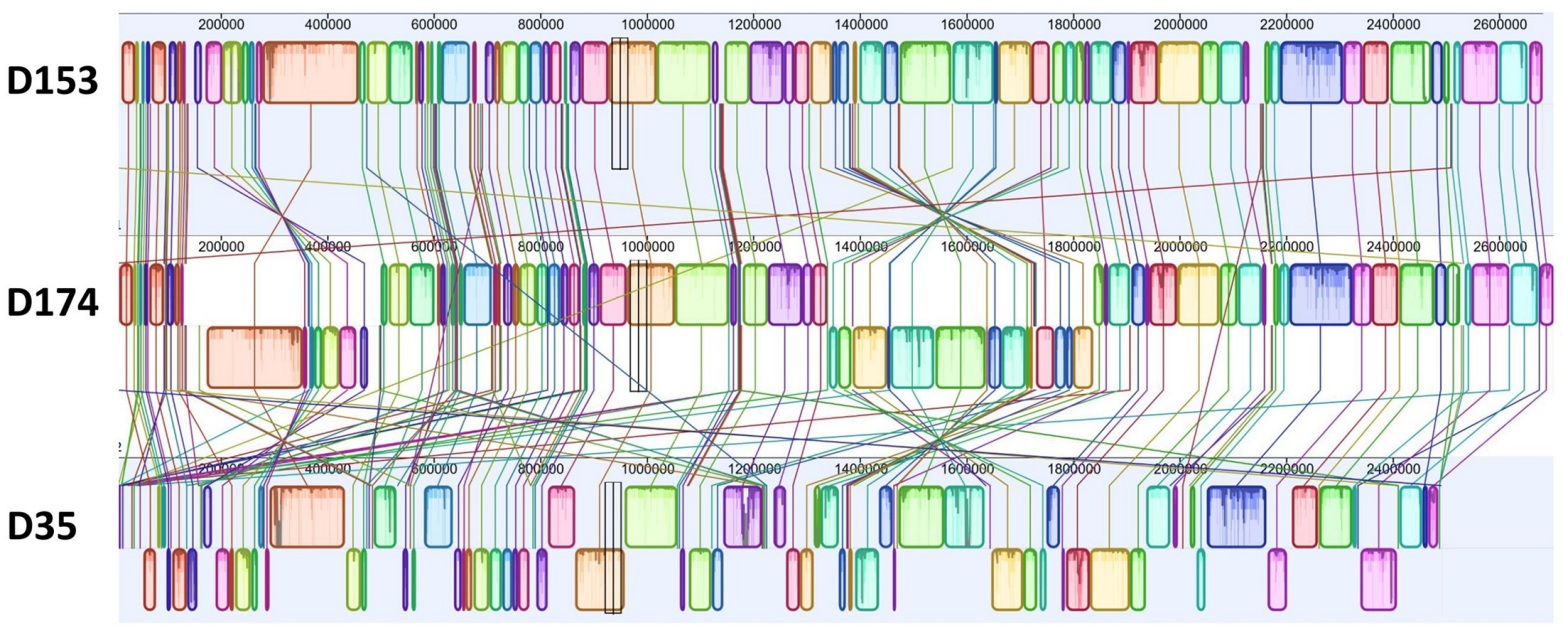
Schematic diagrams showing the similarity of the genomes among serotype 1 (D153), serotype 6 (D174), and serotype 2 (D35).

The genome assemblies of *M*. *haemolytica* D153 (St 1) and D174 (St 6) are 2.68 Mb and 2.70 Mb with 2,766 and 2,814 annotated genes respectively [31, 33]. The shotgun sequence of D35 (St 2) was assembled into 123 contigs with a total genome size of 2.49 Mb and 2,513 annotated genes [32]. Alignment of the three serotype genomes by Mauve showed that they shared lots of locally collinear blocks (LCB) but more inverted LCBs for D35 than D174 comparing to D153 (Fig 1) [34]. To compare transcriptome profiles of BF and PL cells among the three serotypes, the D153 genome with median size and number of annotated genes was used as reference for RNAseq data mapping and downstream analysis.

Raw sequencing reads were evaluated by FastQC [35], and cleaned by cutadapt [36]. The complete genome assemblies of D153 (gene bank accession CP005972.1), D174 (gene bank accession CP006574.1), and D35 with 123 draft genome contigs (gene bank accession AUNK00000000.1) were downloaded from NCBI database [32]. The contigs of D35 were concatenated manually based on blast analysis results with D153 genome as the query sequence. The unaligned contigs of D35 were concatenated and placed in front of the aligned contigs. To select a reference genome, the whole genome alignment of the three *M*. *haemolytica* serotypes was done using progressiveMauve algorithm which was plugged into Geneious Prime (2019.2.3) [34].

The cleaned RNA sequences with quality scores of at least 30 were mapped against the reference genomes by STAR [37]. The generated bam file was sorted with SAMtools [38], and counted with RSEM [39]. Raw sequence counts were analyzed using DESeq2 package with Approximate Posterior Estimation for generalized linear model (apeglm) method [40, 41] with criteria of log2 (fold change) greater than 2 or smaller than -2 and false discover rate (FDR) <0.01 as cutoff values to identify differentially expressed genes. Raw RNA-seq data were deposited in NCBI SRA under BioProject accession number PRJNA1008645.

## Results

### Transcript mapping and differentially expressed genes

**Fig 2:**
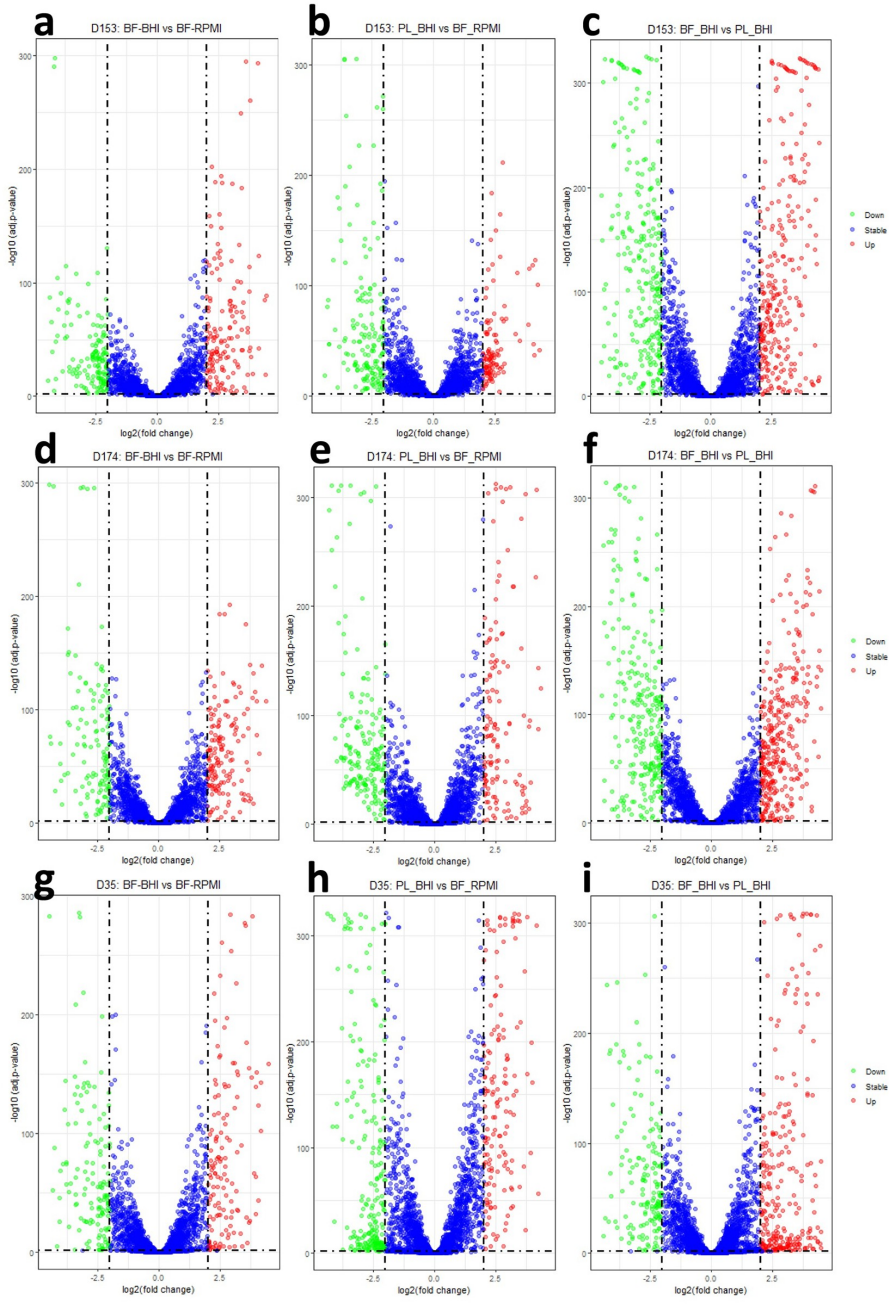
Volcano plot of log_10_ (adjusted p-value) vs. log_2_ fold change shows differentially expressed genes (DEGs) between two groups in each serotype. D153 (St 1) **(a**); biofilm associated cells in BHI vs RPMI **(b)**; biofilm associated cells in RPMI vs planktonic cells in BHI **(c)**; planktonic cells vs biofilm associated cells in BHI. D174 (St 6) **(d)**; biofilm associated cells in BHI vs RPMI **(e)**; biofilm associated cells in RPMI vs planktonic cells in BHI **(f)**; planktonic cells vs biofilm associated cells in BHI. D35 (St 2). **(g**); biofilm associated cells in BHI vs RPMI **(h)**; biofilm associated cells in RPMI vs planktonic cells in BHI **(i)**; planktonic cells vs biofilm associated cells in BHI. Green dots = down DEGs; blue dots = stable DEGs; red dot = up DEGs.

**Fig 3:**
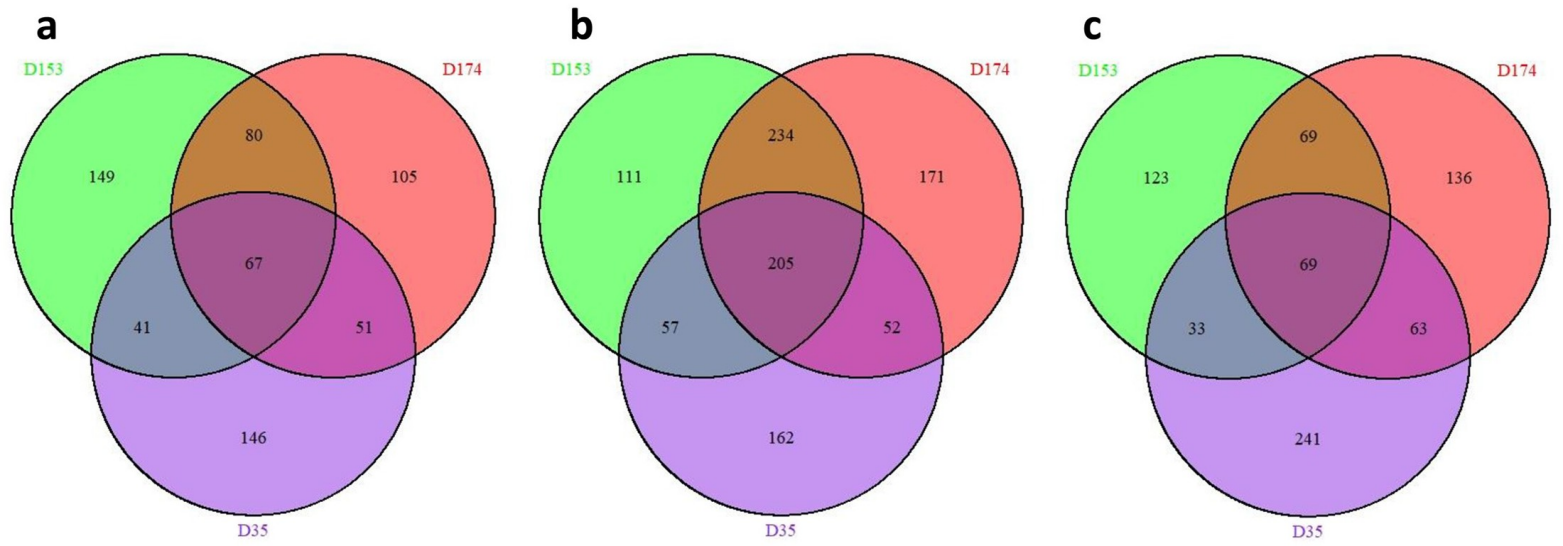
Venn diagrams of the differentially expressed genes. (a); Identified between biofilm associated cells cultured in RPMI vs BHI for St 1 (D153), St 6 (D174), and St 2 (D35) (b); identified in BHI between planktonic cells vs biofilm associated cells for D153, D174, and D35 (c) identified between biofilm associated cells cultured in RPMI vs planktonic cells in BHI for D153, D174, and D35.

Schematic diagrams of the genomes of *M*. *haemolytica* three serotypes are shown in Fig 1. Transcriptome sequencing of the samples produced a total of 1,446,301,986 raw and 1,423,191,551 cleaned sequence reads (S1 Table). The cleaned reads per sample ranged between 14,744,621 and 42,714,703 with average value of 31,626,479 among the samples. Alignment of the cleaned reads uniquely mapped 6,412,192 to 33,043,757 sequences/sample and multiply mapped 1,195,160 to 23,684,672 sequences/sample to the reference genome. Comparisons between the transcriptomes derived from PL cells grown in BHI medium and BF cultured in BHI and RPMI media were done with DESeq2 [40]. The results showed that the highest number of differentially expressed genes (DEGs) were identified by PL and BF cells grown under BHI medium for the three serotypes (Fig 2a–2i; S1 Fig). The distribution of the DEGs in the three serotypes is shown in Fig 3a–3c. The DEGs shared by all three serotypes in BF associated cells cultured in RPMI vs BHI, BF vs PL cells cultured in BHI, and PL cells in BHI vs BF associated cells cultured in RPMI, were 67, 205, and 69 respectively.

### Functional analysis of the DEGs in the biofilm associated cells cultured in BHI and RPMI media

The number of DEGs identified from the cells associated with the BF cultured in BHI vs RPMI media were 337, 303, and 305 for D153, D174, and D35 respectively (S1 Fig). The specific and shared DEGs among the three serotypes are shown in Fig 3a. Although 149, 105, and 146 DEGs were identified uniquely for D153, D174, and D35 respectively, functional analysis of the DEGs indicated that the secondary level of gene ontology (GO) terms in the three GO categories (biological process, molecular function, and cellular component) were very similar among three serotypes (Table 1). Such as in biological process, the DEGs are mainly involved in cellular process, metabolic process, localization, biological regulation, and response to stimulus. Their molecular functions mainly involved in binding, and activities associated with catalytic, transporter, structural molecule, ATP-dependent, and transcription regulator. Many DEGs were identified in cellular anatomical entity and protein containing complex; while in virion component, only one DEG was found for D153, and two for D174.

**Table 1:** Second level of GOs and number of the DEGs identified by comparing biofilm associated cells cultured in BHI and RPMI for D153, D174, and D35.

GO category	GO ID	GO name	Number of DEGs		
			D153	D174	D35
Biological process	GO:0009987	cellular process	140	132	109
	GO:0008152	metabolic process	120	109	100
	GO:0051179	localization	36	42	31
	GO:0065007	biological regulation	30	27	22
	GO:0050789	regulation of biological process	29	25	20
	GO:0050896	response to stimulus	20	19	19
	GO:0048518	positive regulation of biological process	11	5	7
	GO:0048519	negative regulation of biological process	10	7	9
	GO:0032501	multicellular organismal process	6	5	4
	GO:0032502	developmental process	5	5	5
	GO:0000003	reproduction	4	5	4
	GO:0016032	viral process	3	3	2
	GO:0022414	reproductive process	3	4	3
	GO:0042592	homeostatic process	3	3	1
	GO:0044419	biological process involved in interspecies interaction between organisms	2	3	2
	GO:0040011	locomotion	2	1	0
	GO:0002376	immune system process	2	3	1
	GO:0040007	growth	1	0	1
	GO:0098754	detoxification	1	0	2
	GO:0051703	biological process involved in intraspecies interaction between organisms	1	3	0
Molecular function	GO:0005488	binding	113	87	84
	GO:0003824	catalytic activity	111	99	98
	GO:0005215	transporter activity	24	30	21
	GO:0005198	structural molecule activity	15	19	16
	GO:0140657	ATP-dependent activity	9	8	7
	GO:0140110	transcription regulator activity	8	10	5
	GO:0045182	translation regulator activity	5	2	1
	GO:0044183	protein folding chaperone	3	3	1
	GO:0016209	antioxidant activity	3	5	4
	GO:0098772	molecular function regulator activity	2	1	4
	GO:0060089	molecular transducer activity	1	3	0
	GO:0060090	molecular adaptor activity	1	0	0
	GO:0140299	small molecule sensor activity	0	1	0
	GO:0003774	cytoskeletal motor activity	1	0	0
Cellular component	GO:0110165	cellular anatomical entity	149	142	133
	GO:0032991	protein-containing complex	31	40	34
	GO:0044423	virion component	1	2	0

### The difference between biofilm and planktonic cells cultured in BHI medium

**Fig 4:**
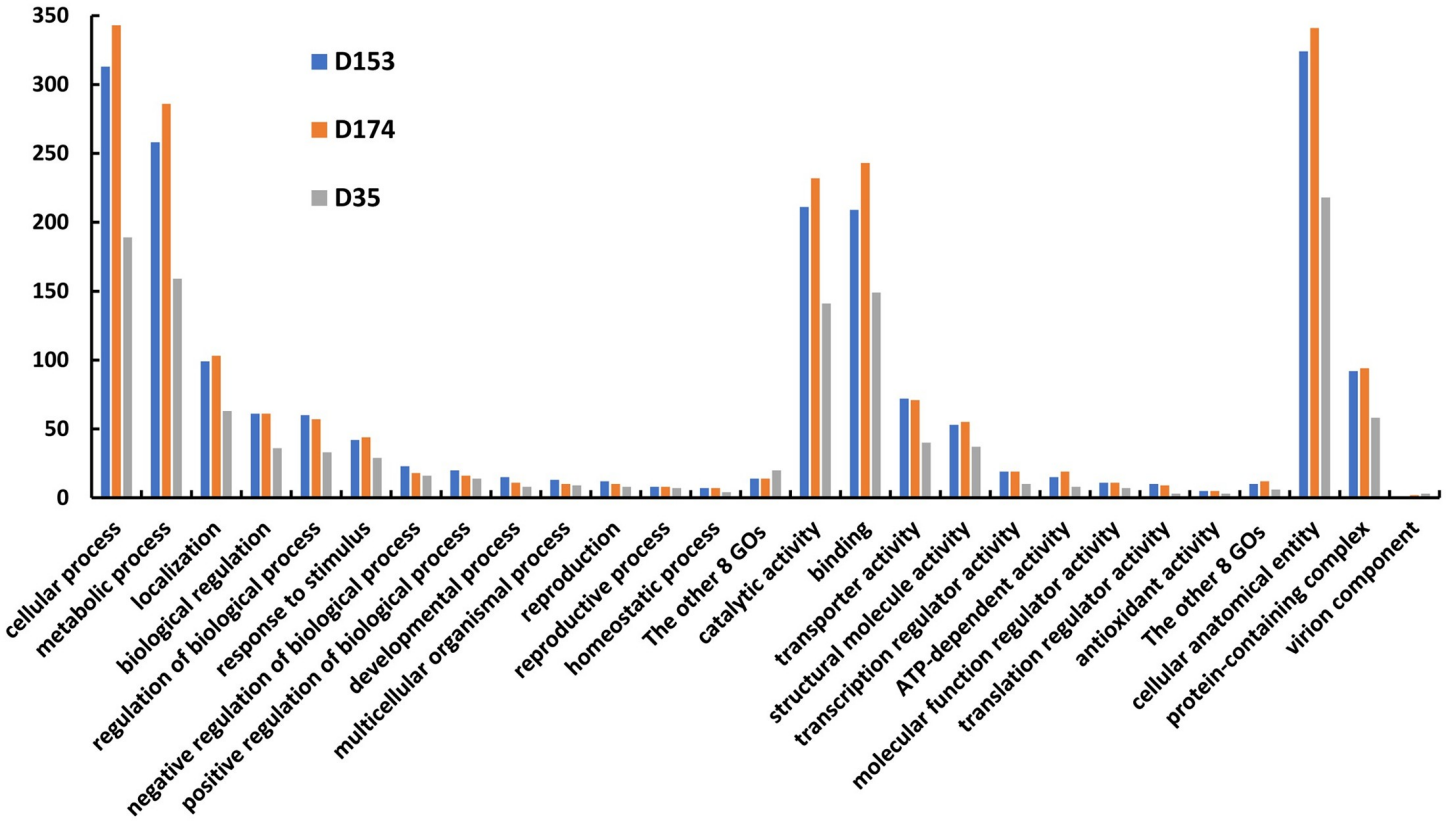
Second level of gene ontology and associated differentially expressed genes identified by comparing planktonic and biofilm cells cultured in BHI for St 1 (D153), St 6 (D174), and St 2 (D35).

Comparing BF and PL associated cells cultured in BHI medium identified 607, 662, and 476 DEGs for D153, D174, and D35 respectively (S1 Fig). A total of 205 DEGs were shared by the three serotypes and 111 to 171 specific DEGs were presented in each of the three serotypes (Fig 3b). The DEGs of the three serotypes have a similar pattern of second level of GOs in the three GO categories (Fig 4). In addition, most of the GOs were the same as that of BF cultured under BHI and RPMI media (Table 1). However, enrichment analysis resulted in significant differences of the enriched GOs of the DEGs identified by BF vs PL cells compared to the DEGs identified by BF cells cultured in BHI vs RPMI media (S2 and S4 Tables). The BF associated cells of the three serotypes shared significantly enriched GOs in translation and peptide biosynthetic process, with the function of structural constituent of ribosome and structural molecule activity. The significantly over enriched GOs in cellular component are mainly related to the ribosome. Six out of seven significantly under enriched GOs identified by BF cells cultured in BHI vs RPMI media were found by BF vs PL cells cultured in BHI medium. Based on the significantly over and under enriched GOs, D153 and D174 are closer among the three serotypes.

**Fig 5:**
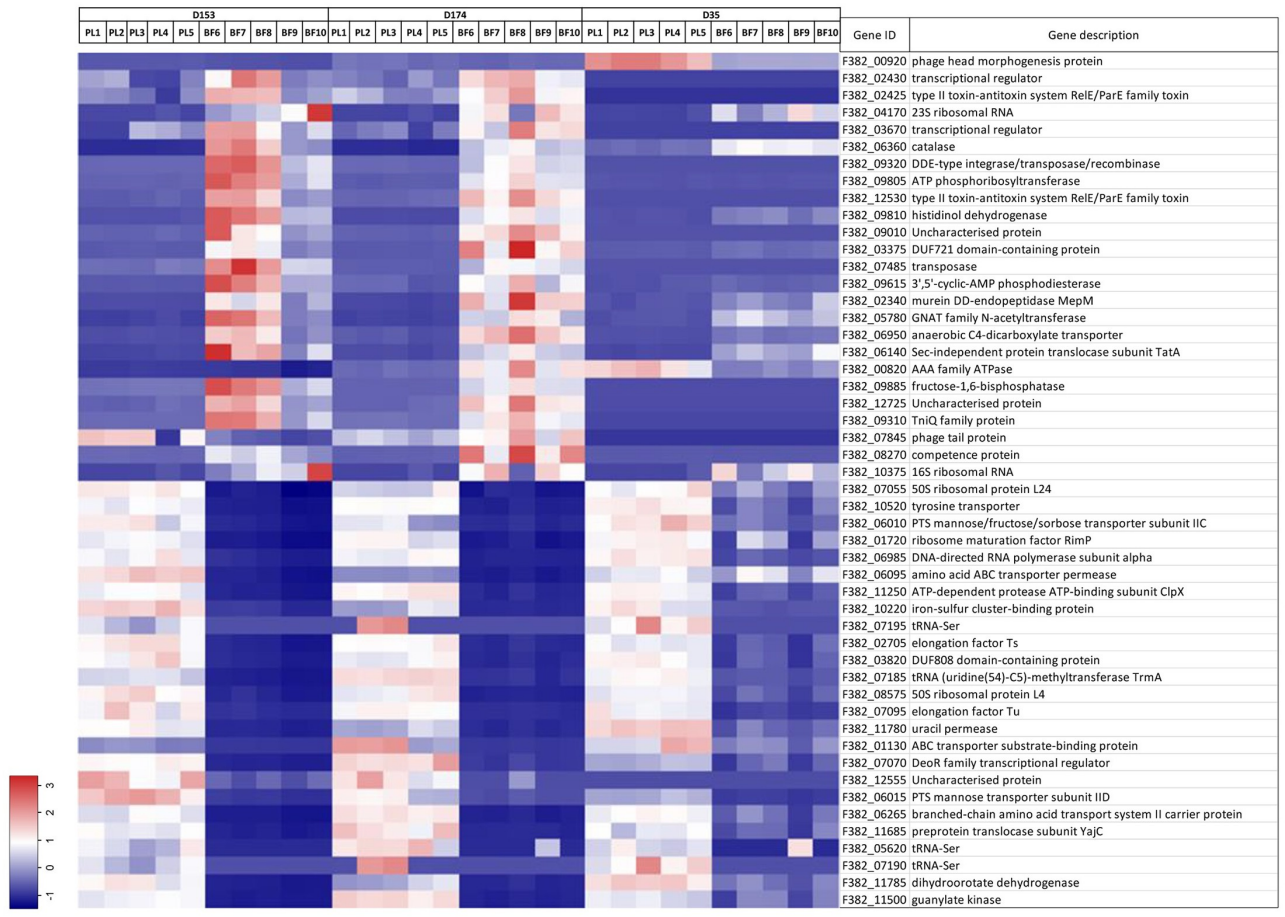
Heat map of differentially expressed genes (DEGs) shared by St 1 (D153) and St 6 (D174) identified between planktonic and biofilm cells in BHI medium. Top 25 up regulated and 25 down regulated DEGs and TPM (transcripts per million) values were shown.

There were 234 DEG specifically shared by D153 and D174, among which 124 DEGs were down regulated and 110 DEGs were up regulated. Top and bottom 25 DEGs based on log_2_ (fold change), and normalized reads (transcripts per million) are shown in Fig 5. The gene ID, gene description, and test results are listed in S5 Table. All significantly enriched GOs of the DEGs were overrepresented (Table 2), which indicated that the expression of the shared genes in BF associated cells were greatly altered comparing to that in PL cells. The significantly enriched GOs of the DEGs are translation, biosynthetic and metabolic processes of amide, peptide, purine, and ATP synthesis. The functions of the DEGs are structural constituent of ribosome, the activities of proton transmembrane transporter, proton channel, and proton-transporting ATP synthase.

**Table 2:** Significantly enriched GOs of the DEGs identified by biofilm cells compared to planktonic cells cultured in BHI medium and specifically shared by D153 and D174.

Tag	GO category	GO term	GO name	Adj. P-value	P-value
Over	Biological process	GO:0043604	amide biosynthetic process	3.54E-02	4.57E-05
Over	Biological process	GO:0006412	translation	3.54E-02	5.13E-05
Over	Biological process	GO:0009206	purine ribonucleoside triphosphate biosynthetic process	3.54E-02	4.91E-05
Over	Biological process	GO:0009145	purine nucleoside triphosphate biosynthetic process	3.54E-02	4.91E-05
Over	Biological process	GO:0043043	peptide biosynthetic process	3.70E-02	6.27E-05
Over	Biological process	GO:0043603	amide metabolic process	3.81E-02	1.01E-04
Over	Biological process	GO:0006518	peptide metabolic process	3.81E-02	8.92E-05
Over	Biological process	GO:0015986	proton motive force-driven ATP synthesis	3.81E-02	1.03E-04
Over	Biological process	GO:0009201	ribonucleoside triphosphate biosynthetic process	3.91E-02	1.22E-04
Over	Biological process	GO:0006754	ATP biosynthetic process	4.81E-02	1.69E-04
Over	Molecular function	GO:0003735	structural constituent of ribosome	3.54E-02	3.78E-05
Over	Molecular function	GO:0015078	proton transmembrane transporter activity	3.54E-02	2.49E-05
Over	Molecular function	GO:0015252	proton channel activity	3.70E-02	7.66E-05
Over	Molecular function	GO:0046933	proton-transporting ATP synthase activity, rotational mechanism	3.70E-02	7.66E-05
Over	Cellular component	GO:0044391	ribosomal subunit	3.54E-02	3.69E-05
Over	Cellular component	GO:0045259	proton-transporting ATP synthase complex	3.86E-02	1.12E-04
Over	Cellular component	GO:1990904	ribonucleoprotein complex	4.08E-02	1.35E-04

### The variations of the three serotypes response to culture medium

The DEGs identified by comparison between BF cells in RPMI and PL cells in BHI for D153, D174, and D35 were 294, 337, and 407 correspondingly (S1 Fig and Fig 3c). Functional analysis of the DEGs resulted the same kinds of GOs in biological process, molecular function, and cellular component as that of the DEGs identified from BF vs PL cells cultured in BHI medium for the three serotypes (S3 Table and Fig 5). This is consistent with above observation that medium didn’t contribute much to the formation of the bacterial colonies. However, the differences among the three serotypes clearly showed by each of them missing some GOs in biological process and molecular functions. There were no enriched GOs for D35, but there were 21 shared GOs significantly enriched between D153 and D174 (S6 Table). This is consistent with above observations that D153 and D174 are closer among the three serotypes based on functional gene analysis.

## Discussion

We and others have previously shown that *M*. *haemolytica* St 1 can form prolific biofilms *in vitro* on the surfaces such as polystyrene tissue culture plates [30, 42] and bovine respiratory (bronchial) epithelial cells [5]. It is our understanding that this is the first study to show St 2 and St 6 strains can also form biofilms *in vitro* very similar to St 1. These observations suggest that *M*. *haemolytica* can form biofilms *in vivo* in order to escape from host defense system. Although one case study demonstrated the presence of *M*. *haemolytica* in biofilm-like microcolonies embedded within bacterial glycocalyx from the pneumonic lung samples, such *M*. *haemolytica* biofilms in tonsil crypts and/or nasal passage are yet to be identified. Pure cultures of *M*. *haemolytica* St 1 and 6 can be recovered from tonsil swabs and washes however, we could not demonstrate the presence of biofilms despite detecting bacteria in H&E slides even when using ideal fixatives for biofilms (Carnoy’s and Methacarn; unpublished observations). Since multiple up-regulated genes were identified in BF compared to PL cells, some of these genes could be used as a potential marker to identify *M*. *haemolytica* BF from the tonsils.

Biofilm is a self-generated extracellular materials assembly that produces a specialized microenvironment which endows microbes with increased resistance to antibiotics and immune defenses [7, 9, 12, 18]. The comparison of DEGs between BF and PL associated cells can provide important information on what genes are differentially expressed under two growth conditions. In this study, we analyzed the transcriptomic libraries prepared from *M*. *haemolytica* St 1, 2 and 6 cultured under PL and BF growth conditions. Comparison between BF and PL associated cells cultured in BHI medium identified large number of DEGs under stringent criteria and higher transcripts expression was noticed for BF as compared to PL associated cells. Those DEGs are mainly related to biological process such as cellular and metabolic processes, localization, biological regulation, and response to stimulus with molecular functions of binding, and activities of catalytic, transporter, structural molecule, and transcription regulator. The cellular components of the DEGs were cellular anatomical entity and protein-containing complex. Because the culture times were limited to 24 hours without shaking, the DEGs of the three *M*. *haemolytica* serotypes cultured in BHI medium likely were in the growth stage of biofilm development [14]. However, the dynamics of the biofilm life cycle may vary across the different serotypes and media examined here. To that point, for an example, *M*. *haemolytica* St 1 (D153) was shown to highly express the carbon storage regulator gene (Gene ID: F382_01930) in BF cells grown in BHI medium when compared to BF cells in RPMI medium. This suggest that BHI may enhance BF maturation earlier than RPMI for D153, and that D153 may have started to prepare for disaggregation of bacteria from the biofilm, as the gene product promotes biofilm dispersal in *E*. *coli* [43].

By comparing biofilm cultures in BHI and RPMI media, the 2^nd^ level of GOs had very similar trends of the DEG numbers, but only D35 had some significantly enriched GOs (S4 Table). Alternatively, by comparing BF cells cultured in RPMI medium, and PL associated cells cultured in BHI medium, the significantly enrich GOs were only identified in D153 and D174, but not in D35 although the 2^nd^ level of GOs had a similar trend of the associated DEG numbers (Table 1 and S3 Table). Consistent with previous report, our GO analyses results showed that strains D153 and D174 are more closely related to one another than to D35 [44]. These results strongly support the immunological and molecular genetic studies on *M*. *haemolytica* serotypes [43, 45–49]. The leukotoxin of *M*. *haemolytica* is the major virulence factor involved in the development of BRDC. Surprisingly, we did not observe any up or down regulation of leukotoxin genes (*lktC*, *lktA*, *lktB* or *lktD*) either from PL or BF associated cells. However, up regulation of several type II toxin-antitoxin system RelE/ParE family toxins (F382_00510; F382_03405; F382_05365; F382_07655; F382_12530) were observed with BF compared to PL cells (S5 Table).

*M*. *haemolytica* serotype surveillance of commercial feedlot in western Canada reported that St 2 strains were commonly isolated from healthy cattle, and St 1 or St 6 were more often isolated from individuals with BRD [27]. Another study in the Netherlands reported that St 1 and St 6 strains were predominantly isolated from adult dairy cattle with acute fibrinous pleuro-pneumonia while St 2 strains were isolated from veal calves with acute fibrinous polyserositis [25]. Several studies have also reported that St 1 and St 6 are most common in sick cattle [23, 26, 28, 29]. Although St 2 is more frequently isolated from healthy cattle, it can cause polyserositis in calves [25] and pneumonia in adult cattle. Some of the DEGs identified and shared by St 1 and St 6 might be important factors related to BRD, especially the genes of adhesins which contain immunodominant domains and could be explored as candidates for making engineered vaccine strains against BRD.

*M*. *haemolytica* St 1 expresses several outer membrane proteins (OMPs) of which OmpA has been involved in the attachment to respiratory (bronchial) epithelial cells and biofilm formation [42, 50, 51]. Cattle vaccinated with *M*. *haemolytica* St 1 specific OMPs such as SSA-1 and OmpA stimulated higher antibody response compared to cattle vaccinated with OmpP2 and OmpD15 [52]. Consistent with those findings, our results also showed that the membrane protein gene *ompA* (F382_08755) was differentially expressed in PL cells compared to BF cells cultured in BHI medium for D153 and D174, but this DEG was not differentially expressed in D35 (S5 Table). This observation further suggests that recombinant OmpA may be a suitable target protein to include with *M*. *haemolytica* vaccines to improve protective vaccine potential [53]. It is also noteworthy to highlight that differentially expressed elongation factor Tu gene (F382_07095) in St 1 and St 6 identified in this study could also be a potential vaccine target as this protein has been shown to produce not only systemic antibody response but also protect calves against *Mycoplasma bovis* challenge [54].

Biofilm formation has been divided into five-step biofilm model [14]. Within each step, the phenotypic variations may occur. For example, extensive variation in colony morphology for *Pseudomonas aeruginosa* was reported when 2–7 days old biofilms were grown on standard agar [55]. The degree of variation in colony morphology was increased with the duration of biofilm growth. However, such characterization of *M*. *haemolytica* biofilm formation and steps are yet to be performed. Therefore, to understand more about *M*. *haemolytica* biofilm life cycle, the samples from each stage should be collected and analyzed. In addition, some small RNAs involved in biofilm development have been identified as important regulators in biofilm formation of *P*. *aeruginosa* and *Salmonella enteritidis* [56–59]. It would be interesting to include small RNAs in future biofilm studies of *M*. *haemolytica*.

## Conclusions

In cattle, *M*. *haemolytica* is commonly found in the upper respiratory tract and moves down into the lung when hosts’ immune system is compromised to develop BRD. *M*. *haemolytica* in a biofilm show increased resistance to several antibiotics (gentamicin, florfenicol, and tulathromycin) as compared to PL cells [42]. In this study, although many DEG were shared by the three serotypes, the differences of St 1 (D153) and St 6 (D174), and St 2 (D35), were revealed by the significantly enriched GOs associated with the DEGs. The shared DEGs by D153 and D174 could be important candidate genes for new vaccine development against biofilm formation and BRD.

## Supporting information

S1 Fig Differentially expressed genes identified from comparisons of biofilm associated cells and planktonic cells cultured in BHI and RPMI media for St 1 (D153), St 6 (D174), and St 2 (D35). (TIF)

S1 Table Sequencing and mapping statistics. (XLSX)

S2 Table Significantly enriched gene ontology (GOs) associated with the differentially expressed genes (DEGs) identified from biofilm vs planktonic cells cultured in BHI medium for the three serotypes. (XLSX)

S3 Table Second level of gene ontology (GOs) and the number of associated differentially expressed genes (DEGs) identified by comparing PL cells cultured in BHI and biofilm cells cultured in RPMI for the three serotypes. (XLSX)

S4 Table Significantly enriched gene ontology (GOs) of the differentially expressed genes (DEGs) identified by comparison between biofilm vs planktonic cells cultured in BHI medium for D35 serotype. (XLSX)

S5 Table Differentially expressed genes (DEGs) shared by D153 and D174 and identified by comparison between biofilm associated cells and planktonic cells cultured in BHI medium. Negative fold change denotes increased expression in planktonic cells compared to biofilm associated cells while positive fold change denotes increased expression in biofilm associated cells compared to planktonic cells.
(XLSX)

S6 Table Significantly enriched gene ontology (GOs) associated with the differentially expressed genes (DEGs) identified from biofilm associated cells cultured in RPMI and planktonic cells in BHI. (XLSX)
