# Supplementary figures and images for: Transcriptomic profiles of Mannheimia haemolytica planktonic and biofilm associated cells
Source: PLoS One. 2024 Feb 8;19(2):e0297692. doi: 10.1371/journal.pone.0297692 (PMC10852253; doi:10.1371/journal.pone.0297692)

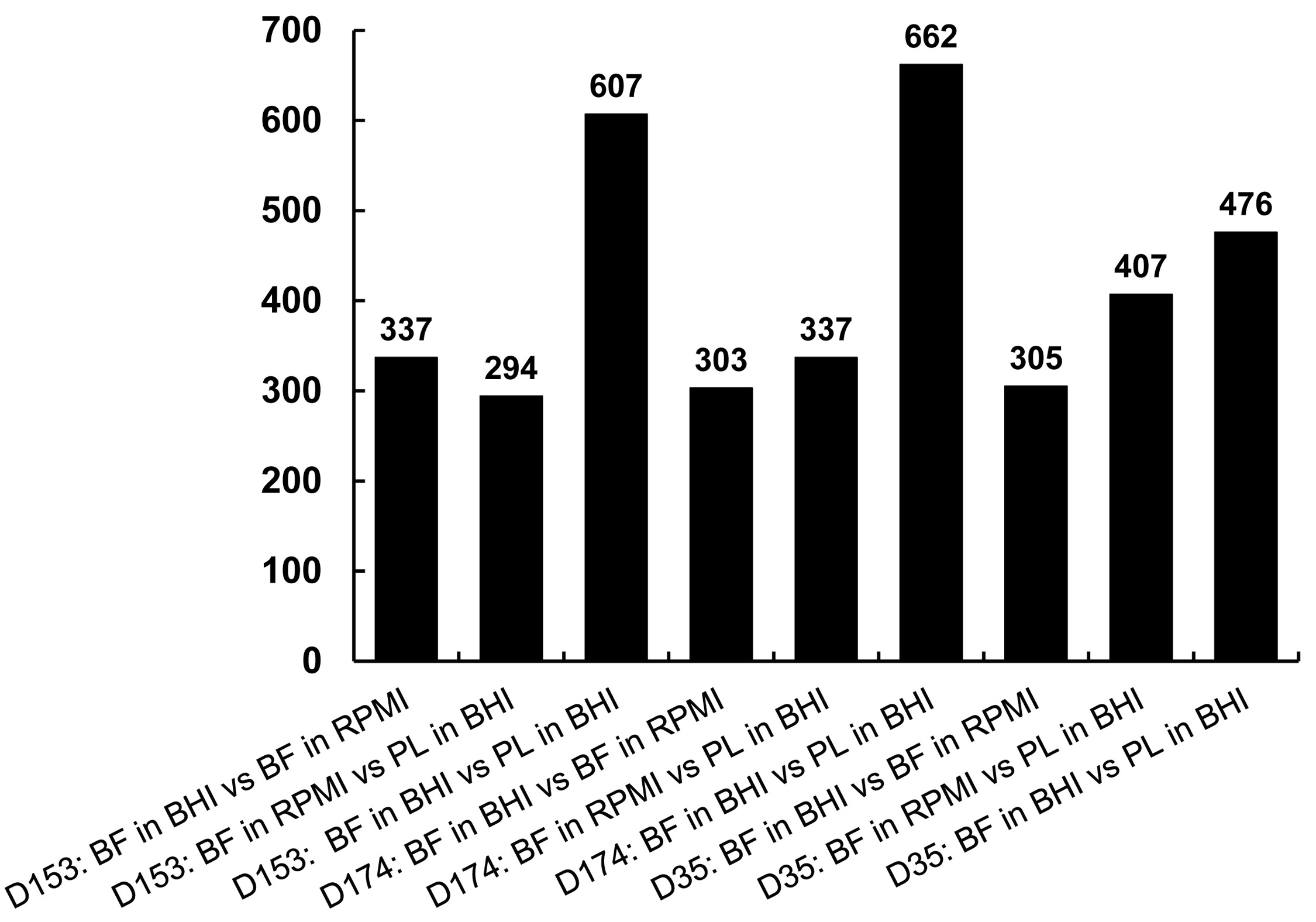

Supplement: S1 Fig — (TIF) [file pone.0297692.s001.tif]
